# AGHE Program of Merit for Gerontology Programs

**DOI:** 10.1093/geroni/igaa057.1737

**Published:** 2020-12-16

**Authors:** Pamela Elfenbein

**Affiliations:** University of North Georgia, Gainesville, Georgia, United States

## Abstract

The POM designation provides gerontology programs with an AGHE “stamp of approval,” which can be used to verify program quality to administrators, to lobby for additional resources to maintain a quality program, to market the program, and to recruit prospective students into the program. This worldwide process of evaluation for both Gerontology programs verifies for students that the program is consistent with globally vetted criteria in gerontology endorsed and recognized by AGHE; assures the public of the quality of programs and their graduates; clarifies for employers the knowledge and skills imparted to students who graduate from POM designated gerontology programs; informs campus administrators of global guidelines, expectations, and practice in gerontology education programs; and indicates to interested students that the program is of high quality. Those interested in the Gerontology Program of Merit will be able to ask questions, understand the application process, and ask for technical assistance.

